# Staufen1s role as a splicing factor and a disease modifier in Myotonic Dystrophy Type I

**DOI:** 10.1080/21675511.2016.1225644

**Published:** 2016-08-19

**Authors:** Emma Bondy-Chorney, Tara E. Crawford Parks, Aymeric Ravel-Chapuis, Bernard J. Jasmin, Jocelyn Côté

**Affiliations:** Department of Cellular and Molecular Medicine, University of Ottawa, Center for Neuromuscular Disease, Ottawa, Ontario, Canada

**Keywords:** alternative splicing, Alu elements, disease modifiers, Myotonic Dystrophy Type I, pre-mRNA splicing, Stau1-binding sites, Staufen1

## Abstract

In a recent issue of *PLOS Genetics*, we reported that the double-stranded RNA-binding protein, Staufen1, functions as a disease modifier in the neuromuscular disorder Myotonic Dystrophy Type I (DM1). In this work, we demonstrated that Staufen1 regulates the alternative splicing of exon 11 of the human Insulin Receptor, a highly studied missplicing event in DM1, through Alu elements located in an intronic region. Furthermore, we found that Staufen1 overexpression regulates numerous alternative splicing events, potentially resulting in both positive and negative effects in DM1. Here, we discuss our major findings and speculate on the details of the mechanisms by which Staufen1 could regulate alternative splicing, in both normal and DM1 conditions. Finally, we highlight the importance of disease modifiers, such as Staufen1, in the DM1 pathology in order to understand the complex disease phenotype and for future development of new therapeutic strategies.

## Staufen is a double-stranded RNA binding protein with multiple functions in cytoplasmic RNA handling

**Figure 1.**
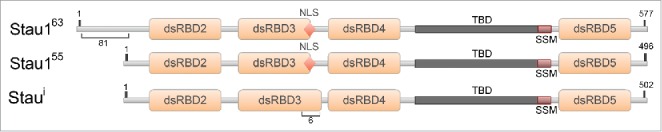
Diagram of the mammalian Staufen1 isoforms. All Stau1 isoforms contain the double-stranded RNA-binding domains (dsRBDs) 2, 3, 4, and 5 (orange boxes), the nuclear localization signal (NLS), the tubulin binding domain (TBD), and the reported Staufen-swapping motif (SSM) ^65^ (red diamond, dark gray and red boxes, respectively). The observed molecular weights are indicated in superscript and the amino acid positions are indicated in numbers.

Staufen proteins are highly conserved, ubiquitously expressed, double-stranded RNA-binding proteins (RBPs).^1,2^ First described in *Drosophila* oocytes, Staufen is essential for anterior-posterior patterning via the localization of the *bicoid* and *oskar* mRNAs.^3,4^ In mammals, there exists 2 homologs of Staufen, Staufen1 (Stau1) and Staufen2 (Stau2). Three major protein isoforms (Stau1^55^, Stau1^63^ and Stau1^i^) are produced from the *Stau1* gene as a result of alternative splicing (Fig. 1). Stau1^55^ and Stau1^63^ bind RNA through their functional RNA-binding domains (RBDs), RBD3 and RBD4, however, it is reported that Stau1^i^ lacks the ability to bind RNA.^2,5^ Staufen proteins, particularly Stau1, are recognized as multi-functional proteins involved in several aspects of cytoplasmic RNA metabolism, such as neuronal transport of RNA, translation efficiency, the stability of specific target mRNAs, and long-term memory formation in *Drosophila*.^6-10^

One aspect of Stau1 biology that research has focused on recently is identifying Stau1 mRNA targets and Stau1-binding-sites (SBSs) where several high-profile studies have improved our understanding of Stau1s multi-functional nature.^10-14^ From these studies, it appears that Stau1 does not have a particular affinity for any RNA-sequence specific based motif(s), but instead prefers short stem structures, similar to the 19 base-pair (bp) stem within the 3′UTR of the *ARF1* gene. Stau1 can also bind secondary structures varying in length and number of perfect base pairing, as well as shorter motifs located within complex structures spanning hundreds of nucleotides, such as those found in 18S rRNA.^8,10,12-14^ One common feature found in the majority of these studies are SBSs containing Alu elements, formed from either one Alu in conjunction with a long non-coding RNA or as an imperfectly paired inverted duplex structure formed from 2 Alu elements in opposite orientation, referred to as Inverted Repeat Alus (*IRAlus*).^13,15,16^ The location of these SBS appear highly variable and many have been reported in 3′- and 5′-UTRs, coding and intronic regions, as well as intergenic regions.^10,13^ From these studies, it could be inferred that Stau1 may be involved in additional functions, potentially including nuclear RNA processing.

## A novel role for stau1 in pre-mRNA alternative splicing

Our lab was the first to provide evidence suggesting a role for Stau1 in the regulation of pre-mRNA splicing.^9^ Such a role for Stau1 has been the subject of much speculation within the field over the years but remained highly debated until recently. For example, one major argument that is often put forth is that Stau1 has been shown to reside primarily in the cytoplasm, which would preclude it from functionally interacting with the nuclear splicing machinery. However, Stau1 is well known to be a shuttling protein as it harbours nuclear localization signal (NLS) sequences and has been observed to localize to the nucleus in several cell types.^17-19^ Moreover, it was shown that a fraction of the Stau1 pool does have a measurable residence time within the nucleus in several cell lines.^19^ More recently, an extensive study carried out by the Moore laboratory that linked transcript secondary structure to the regulation of translation by Stau1, did find that some Stau1 occupancy within intronic regions of transcripts in HEK293T cells, but failed to document any apparent consequence on pre-mRNA splicing.^13^ Nevertheless, work from other groups has revealed additional evidence to support Stau1s role in splicing. For example, an extensive network of splicing proteins was identified by mass spectrometry as components of Stau1 ribonucleoprotein complexes.^20^ A novel role of Staufen protein in splicing was also suggested in *Drosophila* by the Lipshitz laboratory when they observed that alternatively spliced genes were significantly enriched in Staufen targets in *Drosophila* cells.^12^ As a follow up to our 2012 work on Stau1 and splicing, we have recently published a study (Bondy-Chorney et al., *PLOS Genetics*, 2016) where we demonstrate that Stau1 can, in fact, regulate the profile of numerous alternative splicing events (ASEs) in human myoblasts (see below), potentially through interaction with Alu elements present in introns flanking the alternative splicing unit.

## How does stau1 influence alternative splicing?

**Figure 2.**
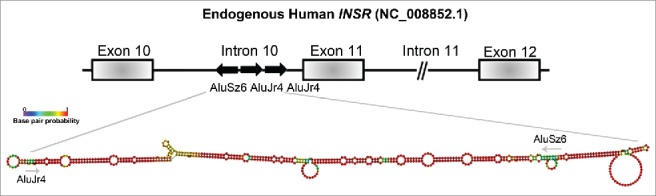
The proposed RNA-secondary structure of the *IRAlus* located in the *INSR* intron 10. The genomic DNA sequence of the human *INSR* (NG_008852.1) was used to assess the Alu repeat elements located in intron 10. Alu elements were identified using RepeatMasker and RNA secondary structure was determined by Vienna package RNAfold 2.1.1. The intronic regions shown here are not to scale and this is indicated by a // symbol.

In order to understand the mechanism behind the alternative splicing regulatory function of Stau1, we focused on the splicing of exon 11 of the human *INSR*, as we previously showed it to be a Stau1-regulated event. Through the modulation of Stau1 via depletion and/or overexpression, we observed that Stau1 regulated the inclusion of exon 11 of the *INSR* through an interaction with Alu elements located in the upstream intron of the alternative event. Prediction of the minimum free energy (MFE) RNA secondary structure of the 3 Alu elements of the *INSR*, using Vienna package RNAfold 2.1.1, revealed that the first and second Alu elements of intron 10 form *IRAlus*, similar to those previously described to be bound by Stau1 (Fig. 2).^13,15,21^ It will be important to generate individual Alu deletion mutants that would disrupt the predicted secondary structure in order to determine whether the formation of this *IRAlus* is necessary for Stau1-regulated splicing of the exon 11. Moreover, insertion of the *INSR IRAlus* sequence upstream of a heterologous alternative splicing cassette would allow for the investigation into whether this induces Stau1-regulated splicing of said exon. These experiments would be crucial to study the Alu-directed Stau1 splicing mechanism on the *INSR* and other Stau1 alternative splicing targets.

Next, when investigating the role that the Alu elements located in intron 10 of the *INSR* have on splicing of exon 11, we can hypothesize, based on our work and previous literature, the type of cis-acting elements they form. For instance, we demonstrated in our recent paper, that the absence of intronic Alu elements resulted in an increase in exon 11 inclusion, a finding also reported in HepG2 cells.^22^ Thus, we could speculate that the presence of *IRAlus* serve to inhibit the inclusion of exon 11, suggesting that these elements act as Intronic Splicing Silencers (ISS). Finally, although it appears that Stau1 binds directly to the Alu elements, in some form of higher order RNA-secondary structure, we cannot exclude the participation of additional RNA-binding proteins that could provide some sequence specificity. It is widely accepted that the mechanisms involved in regulation of alternative splicing is often very complex and commonly involve several trans-acting factors and cis-acting elements. For example, if we hypothesize that the Alu elements are acting as ISS, perhaps once Stau1 is bound to these elements this leads to the recruitment of a trans-acting factor that may interfere with recruitment of constitutive splicing factors, resulting in a reduction of exon splicing. It is likely that Stau1 interacts with other splicing proteins in order to mediate its regulation of alternative splicing. Intriguingly, previous studies have predicted that Stau1 interacts with numerous splicing proteins in Stau1 ribonucleoprotein complexes, including SFRS1, PRPF8, SF3B1, SF3B2, hnRNP M, hnRNP U, and hnRNP C.^20^ The interaction between Stau1 and hnRNP C may be of particular interest, in terms of Stau1-regulated splicing as it was previously demonstrated that hnRNP C interacts with Alu elements to regulate hundreds of splicing events.^23^ Interestingly, Zarnack and colleagues further demonstrated that hnRNP C competes with the splicing factor U2 snRNP auxiliary factor 65-kDa subunit (U2AF65), thereby preventing its binding to Alu elements.^23^ Elucidating the nature of the interactions between Stau1 and other splicing proteins in specific tissues, using, for example, quantitative proteomics approaches, would help define further the Stau1 splicing regulatory mechanism. Additional studies will be required in order to fully decipher the specific details of how Stau1 regulates alternative splicing and how broadly applicable it can be.

## Stau1 is misregulated in myotonic dystrophy type I

DM1 is an autosomal dominant neuromuscular disorder caused by an expansion of CTG-repeats in the 3′ untranslated region (3′UTR) of the dystrophia myotonica protein kinase (DMPK) gene.^24^ Once transcribed, the CUG-repeats of the *DMPK* mRNA form hairpin-like secondary structures, causing the mRNA to aggregate, which are trapped in the nucleus forming ‘toxic’ RNA foci, which results in misregulation and/or sequestration of several RNA-binding proteins, including transcription factors and importantly, numerous splicing factors. The misregulation of these splicing factors plays a central role in the DM1 pathology through wide-spread missplicing (discussed in detail below).^25-33^ The effect of the CUG-repeats has also been shown to induce posttranscriptional silencing of specific genes through Dicer processed short (CUG) RNAs, misregulation of alternative polyadenylation events and deregulation of specific microRNAs and altered expression of target transcripts.^34-36^ Furthermore, recent reports suggest that repeat-associated non-ATG translation (so-called “RAN translation”) occurs in DM1 CAG expansion transcripts to produce toxic homopolymeric (polyglutamine) proteins that may contribute to the DM1 pathogenesis^37,38^

In our 2012 report, we found that the overexpression of Stau1 was able to reverse key splicing defects, for example, the missplicing of exon 11 of *INSR* and the intronic retention event in the CLC1 pre-mRNA, in the neuromuscular disorder Myotonic Dystrophy Type 1 (DM1). We also observed that Stau1 is naturally upregulated in DM1 skeletal muscle obtained from 3 different DM1 mouse models, and human DM1 muscle biopsies.^9^ Importantly, we observed that Stau1 interacted with CUG-repeat mRNA in a length-dependent manner although it was not stably recruited to RNA foci in DM1 cells. Furthermore, we uncovered that by overexpressing Stau1 we enhanced the nuclear export and protein translation of the CUG-repeat mRNA both in DM1 cell culture and mouse models, an effect that was dependent on Stau1s dsRBD3 and NLS.^9^ Our findings thus prompted us to assess further whether Stau1 might represent a valid therapeutic target for DM1.

As an initial step toward that goal, it was important to establish how broad the effect of Stau1 was on alternative splicing in the context of DM1. Our recent study published in *PLOS Genetics* provided crucial insights on this, as mentioned above, by carrying out a high-throughput RT-PCR screen to investigate hundreds of splicing events, pre-selected as relevant to muscle physiology and function^39^, in wild-type (WT) and DM1 myoblast cells. Briefly, either GFP, used as a CTRL, or Stau1-HA was overexpressed in MyoD-converted WT and DM1 myoblasts and the changes in the splicing patterns between the different conditions was assessed. This type of analysis allowed for us to both identify splicing events that are potentially regulated by Stau1 and additional novel DM1 splicing events in a severe DM1 myoblast cell line containing 1700 CUG-repeats. Using this approach, numerous splicing events were identified that were altered upon Stau1 overexpression in WT and DM1 conditions. In total, using a cut-off of ≥ 10% in percent splicing change (PSI), it was found that Stau1 altered the splicing patterns of 75 and 88 splicing events in WT and DM1 cell lines, respectively. Examples of these Stau1-regulated splicing events were confirmed in the *INSR, hnRNP A2B1, LRRC23, HIF1*α, *NRG1, FN1, ACCN3, FHL3, G6PC3, CLCN2* and *CLCN6* mRNAs. Moreover, by comparing Stau1-regulated splicing events to ones found to be regulated by the well-known splicing regulators MBNL1 and RBFOX1 using the same RT-PCR array, we were able to reach a number of conclusions. For one, the fact that Stau1 influenced a similar proportion of ASEs than those 2 splicing factors provided support for the notion that Stau1 is indeed a bona fide splicing regulator. Second, several splicing events were found to be co-regulated between the 3 splicing regulators, but this overlap was by no means absolute, suggesting Stau1 has its own set of ASEs that it specifically regulates. Lastly, among overlapping targets, there were a balanced proportion (∼60/40) of ASEs where Stau1 was influencing splicing decisions either in the same way or in the opposite direction as either MBNL1 or RBFOX1. This suggests that Stau1 may function as an agonist or antagonist to other splicing factors, and further studies will be needed to fully understand the complexity of the splicing network regulated by Stau1.

These observations also prompted us to assess what impact Stau1 overexpression might have in the context of the DM1 pathology. By assessing whether Stau1 overexpression shifted a splicing event either toward or away from the WT splicing patterns, we were able to determine if Stau1 overexpression would be predicted as being overall beneficial or detrimental for DM1 patients. This was an intriguing notion to us, as we had previously demonstrated that overexpression of Stau1 had rescued 2 key hallmarks of the DM1 phenotype, the aberrant splicing of 2 missplicing events and the nuclear export and translation of CUG-expanded mRNA.^9^ The results of our RT-PCR screen revealed that the overexpression of Stau1 in DM1 resulted in both beneficial splicing events (25 ASEs), such as the rescue of the *INSR* exon 11, and detrimental splicing effects (8 ASEs), which could exacerbate the DM1 pathology, for example, the splicing of *hnRNP A2B1*. Mammalian hnRNP A2B1 is a known splicing factor that produces 2 mRNA isoforms, A2 and B1 as a result of alternative splicing of the 36 bp exon 2. Our splicing screen and validation showed a trend toward increased *hnRNP A2B1* exon 2 skipping in severe DM1 myoblast cells, as compared to WT, and that Stau1 regulates this event. The *hnRNP B1* mRNA, which lacks exon 2, has been shown to be increased in lung cancer tissues and is suspected to be involved in early-stage carcinogenesis.^40^ The hnRNP B1 protein was also shown to interact and inhibit the activity of DNA-dependent protein kinase (DNA-PK).^41^ We envisage Stau1 as a potential modulator of this splicing event in DM1, as endogenous Stau1 is naturally elevated in DM1, and speculate that this could contribute to the DM1 pathology based on the previous literature describing the impact of hnRNP B1 expression in disease conditions. Collectively, the results of our screen reveal the widespread effect that Stau1 has on alternative splicing and also highlight its role as a disease modifier in DM1.

## Stau1 acts as a disease modifier in DM1

**Figure 3.**
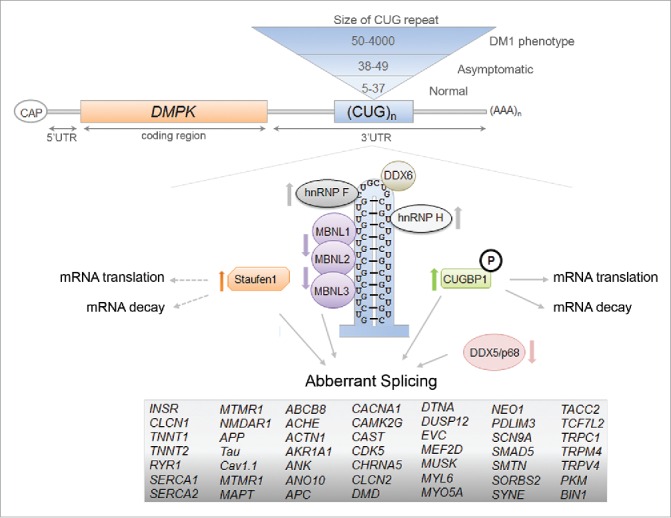
The RNA-binding proteins that are involved in the molecular pathomechanisms of Myotonic Dystrophy Type 1. The toxic RNA-gain of function model of DM1 that shows the expansion of CUG-repeat mRNA in the nucleus and the resulting misregulation of RBPs. The RBPs that can act as disease modifiers in the DM1 pathology, through the regulation of alternative splicing events, mRNA translation and decay, are shown here and the arrows represent the decrease/increase in either protein levels and/or activities of the protein in DM1 (references within main text). The mRNAs containing aberrant splicing events that have been identified in various DM1 models are listed.

In addition to Stau1, there are numerous other misregulated RNA-binding proteins that may act as disease modifiers in DM1 and play an important role in the pathology. Perhaps the most studied are members of the muscle-blind protein family (i.e. MBNL1-3), which have been proposed to be responsible for the majority of the known missplicing events in DM1.^29^ MBNL1 has been found to directly bind to the CUG-repeats and is sequestered by the RNA foci in the nucleus causing MBNL1 loss-of-function resulting in multiple aberrant alterative splicing events in the pathology.^27,42^ Other misregulated RNA-binding proteins in DM1 include CUGBP1, hnRNP H, RNA helicase p68/DDX5, DEAD-box helicase DDX6, TBPH, and BSF (Fig. 3).^43-47^ The misregulation of many of these RBPs has been shown to result in aberrant splicing of pre-mRNAs in DM1. For example, Paul et al. reported that not only were the steady-state levels of hnRNP H, a known splicing regulator, increased in DM1 myoblasts, but that hnRNP H overexpression in myoblasts inhibited *INSR* exon 11 inclusion, similar to the splicing pattern seen in DM1 conditions.^46^ Recently, Jones et al. demonstrated that the DEAD-box RNA helicase, DDX5/p68, was reduced in DM1 skeletal muscle.^43^ They also found that an increase in DDX5/p68 reduced skeletal muscle myopathy and atrophy in a DM1 mouse model and degraded mutant CUG RNAs. Additionally, DDX5/p68 has been shown to allow for increased MBNL1 binding to the mutant CUG-expanded mRNA repeats which, in turn, can influence splicing events misregulated in DM1 as described for the cardiac Troponin T (*TNNT2*) pre-mRNA.^48^ The emergence of these other RBPs that are misregulated in DM1 highlight the complexity of the pathology and it is crucial to identify more factors involved to obtain a better understanding of the disease.

Since the majority of DM1 research has heavily focused on a few key RBPs, such as MBNL1, it may appear easy to attribute the pathology to the misregulation of just a few proteins. This idea should be accompanied with caution for several reasons. For example, although multiple DM1 mouse models have been generated, including MBNL1 and MBNL2 loss-of-function mice, these models fail to recapitulate all of the features of the DM1 pathology in humans.^49,50^ Although it is clear that proteins such as MBNL1 are major players in DM1 pathogenesis, we hypothesize that other RBPs such as Stau1 play important functions as disease modifiers, where the fine-tuning of such genes likely contribute together to the disease progression and severity. The complex phenotype observed in DM1 is likely a cumulative effect of several of these disease modifiers, in addition to epigenetic factors, and environmental influences. The “normalization” of misregulated RBPs is recognized as one of the major approaches being explored in pre-clinical studies in DM1.^32^ It is vitally necessary to broaden the scope of the DM1 disease mechanism to include these disease modifiers and their effects on several aspects of the disease to better understand its complexity. The importance of this notion becomes apparent when potential therapies, for example, the overexpression of MBNL1, are suggested to treat DM1 patients. We speculate that even if the major players, such as MBNL1 are corrected, other disease modifiers misregulated in DM1 such as Stau1, may continue to contribute to the pathology, perhaps through its role in alternative splicing. Indeed, this idea may be reflected in the evidence that although Mbnl1 overexpression, in the widely used DM1 mouse model the HSA^LR^ mouse, was reported to rescue myotonia and several key splicing events known to be misspliced in DM1, other features of the phenotype were not restored, such as normal myofiber structure.^51^ It would be interesting to investigate how the correction of MBNL1 sequestration, and the release of MBNL1 from the CUG-expanded mRNA, would influence the activity of the other disease modifiers bound to the CUG-repeats. Although Stau1 is not stably recruited, it is still possible that the transient interactions between Stau1 and the CUG repeat mRNA may be sufficient to disrupt binding of sequestered RBPs such as MBNL1. If this occurs, then Stau1 upregulation in DM1, may also indirectly regulate some MBNL1-specific splicing events through the displacement of MBNL1 from the CUG-repeats upon Stau1 binding. In order to begin to address these types of ideas we need to further understand the impact of the other disease modifiers that are involved in DM1, for example, Stau1s role as a splicing regulator, and how it contributes to the DM1 pathology.

The importance of disease modifiers has been highlighted for other neuromuscular disorders. For example, the main causative event in Spinal Muscular Atrophy (SMA) a functional loss the *SMN1* gene, however, it has been appreciated for some time now that the *SMN2* gene is a strong modifier of the SMA phenotype, as copy number of this gene closely correlates with disease severity.^52^ More recently, additional disease modifiers have been identified in SMA, for example, the Actin-Binding Protein Plastin 3, whose expression can modify the phenotype of female SMA patients.^53^ In the recessive X-linked form of muscular dystrophy, Duchenne Muscular Dystrophy (DMD), it was recently found that the gene encoding the Latent Transforming Growth Factor-β Binding Protein 4 (LTBP4) whose function is to bind Transforming Growth Factor Beta (TGFβ), was indeed a modifier in DMD.^54^ Taken together, results of these studies indicate that in addition to primary defects, neuromuscular disease severity and progression can be markedly influenced by a host of secondary modifying proteins that should be considered when devising therapeutic approaches for patients. Moreover, as in the case of Stau1 in DM1, modulation of these disease modifiers may even represent attractive therapeutic targets in some cases.

## Outstanding questions and concluding remarks

The recent efforts to understand the multi-functionality of Stau1, for instance, our work into the novel role of Stau1 in splicing and in DM1, has greatly extended the understanding of Stau1 and also brought to light many new considerations. For example, we have previously uncovered a significant upregulation of Stau1 in DM1 skeletal muscle, however, its expression in other DM1 tissues is unknown. It would be interesting to investigate the expression of Stau1 in other tissues affected in DM1 such as the heart and brain. Furthermore, if we do see the overexpression of Stau1 in other DM1 tissues, it would be important to evaluate the alternative splicing of known Stau1 targets in addition to investigating novel splicing events. Several of the Stau1-regulated mRNAs we identified also harbour splicing events shown to be misspliced in the hippocampus of adult DM1 mouse models, for example, *KCNMA1* and *CACNA1d*.^55^ Our recent work shows that Stau1 acts as a splicing regulator in several different cell types, thus, one could speculate that indeed a misregulation of Stau1 in various tissues types, in addition to muscle, would result in splicing changes within these tissues. In addition to Stau1, it would be interesting to examine the expression pattern of Stau2 in various DM1 tissues. As both Stau1 and Stau2 have been shown to play important roles in neuron function^56,57^, assessment of Stau2 levels in the brain, may prove highly informative and relevant in the context of DM1.

Another outstanding question that emerged from our work is the cause and mechanism involved in the aberrant upregulation of Stau1 in DM1. We could speculate on several alternate explanations: First, the overexpression of Stau1 may be a direct result of the CUG-repeat expansions. We have observed a tendency for a disease severity-dependent increase in Stau1 levels in DM1 patient biopsies.^9^ If Stau1 upregulation is a direct result of the CUG-repeat mRNA, then investigating whether Stau1 is overexpressed in other DM1 tissues, where the toxic RNA foci are present, should also reveal an increased Stau1 expression in these tissues. Alternatively, it is possible that among the numerous proteins misregulated in DM1, some of these factors regulate Stau1 expression. Investigating this avenue would be highly informative as little is known about the general regulation of Stau1 mRNA or protein levels. Finally, the aberrant upregulation of Stau1 in DM1 skeletal muscle may not be a direct result of the CUG-repeats rather it may be a consequence of the reversion of DM1 tissues back toward an embryonic state. This reversion toward a neonatal state is observed in skeletal muscle in DM1 and has been suggested as a reason for the adult to embryonic switch in the splicing patterns of several DM1 related splicing events.^58^ It would be interesting to explore whether the aberrant Stau1 upregulation in DM1 contributes to this embryonic shift, perhaps through the regulation of key splicing events. We have previously described Stau1 to be developmentally regulated whereby Stau1 is highly expressed during embryogenesis and decreases to low levels in adult skeletal muscle.^59^ Interestingly, a similar expression pattern is observed for CUGBP1.^59,60^ It is thus possible that several of the misregulated RBPs in DM1, including Stau1, are aberrantly expressed due to the reversion to an embryonic state and, in turn cause missplicing. Finally, many other mechanisms, perhaps independent of the several discussed here, could be contributing to the aberrant upregulation of Stau1 in DM1, thus further investigation is required to fully understand the misregulation of Stau1 in this complex disorder.

Finally, the multi-functional nature of Stau1 should always be considered. Often studies on multi-functional proteins, such as Stau1, are primarily focused on one role of the protein. Due to the multi-functionality of Stau1, it is plausible that in addition to its role in splicing, Stau1 assumes other functions that allow it to further modulate the DM1 pathology. For example, we have demonstrated that Stau1 negatively regulates myogenesis, via the regulation of c-myc translation.^59^ Thus, Stau1 is likely to contribute to the impaired differentiation program observed in DM1.^61^ In addition, Stau1 is recruited to Stress Granules (SGs) to impair their assembly.^62^ Interestingly, we recently reported that SG formation is deficient in DM1 myoblasts, and that this is at least partially due to Stau1 overexpression, as targeting Stau1 using RNA interference rescued normal SGs formation.^63^ Since SGs are part of a protective mechanism for cellular stress, Stau1s negative effect on this process may represent yet another mechanism through which it may act as a disease modifier in DM1. Finally, Stau1 RNP complexes have been reported to contain RNA silencing elements including Ago proteins 1–3 and associated microRNAs, such as miR-124.^64^ Since microRNA deregulation is present in DM1, it would be interesting to investigate whether Stau1 misregulation may also contribute to altered expression of target transcript though microRNA mediated silencing of Stau1-associated microRNAs. These converging lines of evidence thus indicate that Stau1 can act as a disease modifier having widespread effects on several cellular processes that can in turn modulate the DM1 phenotype. Continued research on disease modifiers will advance diagnostic, prognostic and therapeutic avenues necessary to fully understand a complex human disorder such as DM1.

## Abbreviations

3′UTR: 3′ untranslated region
*ACCN3*: Amiloride-sensitive cation channel 3
ASE: alternative splicing events
bp: base-pair
*CACNA1d*: Calcium Channel, Voltage-Dependent, L Type, Alpha 1D Subunit
*CLCN2*: Chloride channel 2
*CLCN6*: Chloride channel 6
DM1: Myotonic Dystrophy Type I
DMD: Duchenne Muscular Dystrophy
DMPK: dystrophia myotonica protein kinase
DNA-PK: DNA-dependent protein kinase
*FHL3*: Four And A Half LIM Domains 3
*FN1*: Fibronectin 1
*G6PC3*: Glucose 6 Phosphatase Catalytic 3
*HIF1*α: Hypoxia-inducible factor 1-α
*hnRNP A2B1*: Heterogeneous Nuclear Ribonucleoprotein A2/B1
*INSR*: Insulin Receptor
*IRAlus*: Inverted Repeat Alus
ISS: Intronic Splicing Silencers
*KCNMA1*: Potassium Channel, Calcium Activated Large Conductance Subfamily M Alpha, Member 1
*LRRC23*: Leucine Rich Repeat Containing 23
LTBP4: Latent Transforming Growth Factor-β Binding Protein 4
MBNL1: Muscleblind-Like 1
MFE: minimum free energy
NLS: nuclear localization signal
*NRG1*: Neuregulin 1
PRPF8: Pre-mRNA Processing Factor 8
PSI: Percent splicing index
RAN translation: Repeat-associated non-ATG translation
RBDs: RNA-binding domains
RBPs: RNA-binding proteins
SBSs: Stau1-binding-sites
SF3B1: Splicing Factor 3b, Subunit 1
SF3B2: Splicing Factor 3b, Subunit 2
SFRS1: Serine/arginine-rich splicing factor 1
SMA: Spinal Muscular Atrophy
Stau1: Staufen1
Stau2: Staufen2
TBPH: TAR DNA-binding protein-43 homolog
TGFβ: Transforming Growth Factor Beta
*TNNT2*: cardiac Troponin T
U2AF65: U2 snRNP auxiliary factor 65-kDa subunit
WT: wild-type.
